# Racial Equity in Healthcare Machine Learning: Illustrating Bias in Models With Minimal Bias Mitigation

**DOI:** 10.7759/cureus.35037

**Published:** 2023-02-15

**Authors:** Michael Barton, Mahmoud Hamza, Borna Guevel

**Affiliations:** 1 Medicine, Harvard Medical School, Boston, USA; 2 Quantitative Methods, Harvard School of Public Health, Boston, USA

**Keywords:** healthcare technology, data science, racial bias, machine learning, health equity

## Abstract

Background and objective

While the potential of machine learning (ML) in healthcare to positively impact human health continues to grow, the potential for inequity in these methods must be assessed. In this study, we aimed to evaluate the presence of racial bias when five of the most common ML algorithms are used to create models with minimal processing to reduce racial bias.

Methods

By utilizing a CDC public database, we constructed models for the prediction of healthcare access (binary variable). Using area under the curve (AUC) as our performance metric, we calculated race-specific performance comparisons for each ML algorithm. We bootstrapped our entire analysis 20 times to produce confidence intervals for our AUC performance metrics.

Results

With the exception of only a few cases, we found that the performance for the White group was, in general, significantly higher than that of the other racial groups across all ML algorithms. Additionally, we found that the most accurate algorithm in our modeling was Extreme Gradient Boosting (XGBoost) followed by random forest, naive Bayes, support vector machine (SVM), and k-nearest neighbors (KNN).

Conclusion

Our study illustrates the predictive perils of incorporating minimal racial bias mitigation in ML models, resulting in predictive disparities by race. This is particularly concerning in the setting of evidence for limited bias mitigation in healthcare-related ML. There needs to be more conversation, research, and guidelines surrounding methods for racial bias assessment and mitigation in healthcare-related ML models, both those currently used and those in development.

## Introduction

Health equity, the ability for everyone to “attain his or her full health potential regardless of socially-determined circumstances,” is one of the most fundamental aims of healthcare and public health [1]. All aspects of a health system landscape - including culture and socioeconomic status, healthcare access and coverage, quality of care, and provider implicit bias - impact the level of health equity in society.

One of the biggest changes in the healthcare landscape has been the rise of machine learning (ML) [2,3]. ML is being progressively incorporated into all parts of healthcare, including the development of diagnostic, clinical prediction, and patient recruitment tools. For example, ML methods have been applied in the prediction of heart failure and various types of cancer [4,5]. These tools have also been involved in the diagnosis of diabetic retinopathy, breast tumors, skin cancer, certain hematological diseases, and even coronary artery disease [6-10]. The power of ML in healthcare continues to grow, and its potential in this setting is vast [2].

While healthcare-related ML is growing more and more powerful in its ability to positively impact human health, the potential for inequity in these methods is concerning. For instance, differing performance and predictive accuracy of ML methods for different social groups can have dramatic implications and further exacerbate health inequities along gender or racial lines. In fact, there have been several studies that have found differing predictive accuracy of ML algorithms by race [11,12]. However, the root causes of the predictive disparities that can occur in ML have not been as well studied. It has been theorized that bias can be introduced at any stage [13]. More specifically, there can be bias involving data collection (e.g., historical bias and measurement bias), data selection (e.g., representation bias), model training (e.g., algorithmic bias), and model deployment (e.g., translational bias) [14].

Opportunities to mitigate potential biases exist at each step of the ML model development pipeline. During the pre-processing stage (before model training), one can reweight training data to increase representation, combine data sets to increase heterogeneity, or even remove race information from the data altogether [15]. During the in-processing stage (during model training), one can use techniques such as regularization or adversarial debiasing [15,16]. During the post-processing stage (after model training), one can calibrate their results or use varying cut-point selections to boost equity in performance [14]. Building ML models with no/minimal bias mitigation techniques can increase the risk of racial model performance disparities.

While ML has seen a steep rise, evidence suggests that the adoption of bias mitigation has not kept pace. A recent meta-analysis showed that while many healthcare-related ML studies assess for racial bias, some of these studies do not correct for this bias [15]. Also, those that do attempt to correct racial bias may use a limited array of bias mitigation techniques. Additionally, only a small number of studies published their code for bias assessment or debiasing [15].

Given this underwhelming attempt at bias mitigation in healthcare-related ML, in our study, we aim to evaluate the presence of racial bias when five of the most common ML algorithms are used to create models with minimal processing to reduce racial bias. We assessed the following five different ML methods: Extreme Gradient Boosting (XGBoost), random forest, naive Bayes, support vector machine (SVM), and k-nearest neighbors (KNN). Further, we a priori chose healthcare access - one of the most important drivers of health equity - as the outcome of prediction for model creation in our analyses.

## Materials and methods

Dataset

We utilized the Behavioral Risk Factor Surveillance System (BRFSS) 2020 sample for our study [17]. We chose this database for its large sample size (useful for training ML models) and its wide array of variables - including medical, psychological, and social variables. The BRFSS is the largest health survey in the world, collecting data on over 400000 individuals every year in all 50 states, as well as the District of Columbia and three US territories.

Outcome

We a priori chose healthcare access as the outcome for our ML models. We used a single survey question for our measure of healthcare access: “Do you have any kind of healthcare coverage, including health insurance, prepaid plans such as HMOs, or government plans such as Medicare, or Indian Health Service?” Outcome choices were “Yes” and “No”; all other answer choices were grouped into missing values. We coded “No” as “1” and “Yes” as “0,” and hence our analysis would be focused on identifying those without healthcare access.

Predictors

All other survey questions (besides our outcome) were considered as possible predictors for our ML models. We excluded variables from our dataset that were (1) related closely to our outcome, (2) survey components (e.g., time of interview), (3) age or sex-specific (e.g., mammography, prostate-specific antigen, or colonoscopy results), (4) redundant, or (5) having over 50% missing data. Race consisted of the following six categories: White, Black, American Indian or Alaskan Native, Asian, Native Hawaiian or other Pacific Islander, or Hispanic. Individuals whose race was classified as "Other" or "Multiracial" were not included in our analysis.

Missing data

We removed any observations that were missing racial or health access data. For all other variables, we assumed that our data were missing at random and used multiple imputation techniques from the MICE (Multiple Imputation by Chained Equations) package in R to prevent bias from list-wise deletion [18]. Our MICE algorithm used predictive mean matching, logistic regression, and polynomial regression to impute values for our predictors. Additionally, we specified the model to use proportional odds logistic regression as the imputation technique for our ordinal variables.

Assessment of racial bias for each machine learning algorithm

After missing data imputation, we performed a variance analysis and confirmed that no variables had zero variance, which would have interfered with the model-building process. Next, we performed a test-train split stratified by our outcome variable (healthcare access). Given the significant size of our total data, we only used 3% of our total 399896 observations for the training set. We chose a variety of the most common ML algorithms for classification for our study: (1) XGBoost, (2) random forest, (3) naive Bayes, (4) SVM, and (5) KNN. To validate each model, we used k-fold cross-validation with k = 10. We used the CARET (Classification and Regression Training) package in R to build all of our models [19]. We predicted healthcare access (binary classification problem) with our specified list of predictors for our test set (97% of data). We used the area under the curve (AUC) as our measure of performance throughout our analysis - an ideal metric for binary classification problems [20]. We split up our test set into each racial category and then compared AUC values for each race for each ML method predicting healthcare access. Additionally, we bootstrapped this analysis 20 times for each of the five ML algorithms to produce confidence intervals for the AUC performance metric for each race for each algorithm.

## Results

Descriptive statistics of the study population

After data cleaning, our final dataset cumulatively consisted of 399896 observations; 51 predictors remained after variable selection. Variables were excluded if those were (1) related to our outcome (3 variables), (2) survey components (42 variables), (3) age or sex-specific (96 variables), (4) redundant (45 variables), or (5) having over 50% missing data (44 variables). Of our study population, 8.5% reported lacking healthcare access. The most common age groups were 65-69 years (10.4%), followed by 60-64 years (10.3%), and 70-74 years (9.5%). Of note, 54.3% of our study population were female, and 51.7% were married. The racial distribution was as follows: 73.7% White, 7.5% Black, 1.7% American Indian or Alaskan Native, 2.5% Asian, 0.5% Pacific Islander, and 9.0% Hispanic. Table 1 presents the full race-stratified descriptive statistics of our study population (before missing data imputation).

**Table 1:** Race-stratified descriptive statistics of the study population (before missing data imputation) *2.2% of our study population had missing race data

Variables	White (n=294702) (73.7%), n (%)	Black (n=29943) (7.5%), n (%)	American Indian (n=6760) (1.7%), n (%)	Asian (n=10081) (2.5%), n (%)	Pacific Islander (n=1994) (0.5%), n (%)	Other (n=3317) (0.8%), n (%)	Multiracial (n=8267) (2.1%), n (%)	Hispanic (n=36078) (9.0%), n (%)	Overall (N=399896) (100%)*, n (%)
Age (years)									
18-24	14981 (5.1%)	1922 (6.4%)	481 (7.1%)	1421 (14.1%)	241 (12.1%)	155 (4.7%)	960 (11.6%)	4527 (12.5%)	25037 (6.3%)
25-29	12638 (4.3%)	1714 (5.7%)	414 (6.1%)	1083 (10.7%)	204 (10.2%)	177 (5.3%)	690 (8.3%)	3547 (9.8%)	20795 (5.2%)
30-34	14688 (5.0%)	1954 (6.5%)	482 (7.1%)	978 (9.7%)	183 (9.2%)	216 (6.5%)	739 (8.9%)	3635 (10.1%)	23304 (5.8%)
35-39	16623 (5.6%)	2090 (7.0%)	541 (8.0%)	985 (9.8%)	172 (8.6%)	228 (6.9%)	704 (8.5%)	3579 (9.9%)	25407 (6.4%)
40-44	16869 (5.7%)	2238 (7.5%)	506 (7.5%)	864 (8.6%)	160 (8.0%)	226 (6.8%)	665 (8.0%)	3624 (10.0%)	25656 (6.4%)
45-49	18099 (6.1%)	2296 (7.7%)	530 (7.8%)	763 (7.6%)	172 (8.6%)	233 (7.0%)	602 (7.3%)	3238 (9.0%)	26367 (6.6%)
50-54	22231 (7.5%)	2663 (8.9%)	640 (9.5%)	770 (7.6%)	170 (8.5%)	241 (7.3%)	644 (7.8%)	3078 (8.5%)	31014 (7.8%)
55-59	27416 (9.3%)	2878 (9.6%)	694 (10.3%)	639 (6.3%)	164 (8.2%)	304 (9.2%)	672 (8.1%)	2741 (7.6%)	36124 (9.0%)
60-64	32142 (10.9%)	3154 (10.5%)	717 (10.6%)	675 (6.7%)	165 (8.3%)	339 (10.2%)	726 (8.8%)	2376 (6.6%)	41024 (10.3%)
65-69	33484 (11.4%)	3023 (10.1%)	600 (8.9%)	565 (5.6%)	134 (6.7%)	324 (9.8%)	595 (7.2%)	2026 (5.6%)	41480 (10.4%)
70-74	31442 (10.7%)	2386 (8.0%)	481 (7.1%)	493 (4.9%)	105 (5.3%)	257 (7.7%)	515 (6.2%)	1518 (4.2%)	37885 (9.5%)
75-79	22506 (7.6%)	1423 (4.8%)	305 (4.5%)	277 (2.7%)	48 (2.4%)	190 (5.7%)	299 (3.6%)	975 (2.7%)	26535 (6.6%)
≥80	26848 (9.1%)	1619 (5.4%)	233 (3.4%)	329 (3.3%)	47 (2.4%)	296 (8.9%)	341 (4.1%)	890 (2.5%)	31297 (7.8%)
Missing	4735 (1.6%)	583 (1.9%)	136 (2.0%)	239 (2.4%)	29 (1.5%)	131 (3.9%)	115 (1.4%)	324 (0.9%)	7971 (2.0%)
Sex									
Male	134947 (45.8%)	11951 (39.9%)	3055 (45.2%)	5139 (51.0%)	907 (45.5%)	1731 (52.2%)	3872 (46.8%)	16505 (45.7%)	182766 (45.7%)
Female	159755 (54.2%)	17992 (60.1%)	3705 (54.8%)	4942 (49.0%)	1087 (54.5%)	1586 (47.8%)	4395 (53.2%)	19573 (54.3%)	217130 (54.3%)
Marital status									
Married	163949 (55.6%)	9510 (31.8%)	2444 (36.2%)	5502 (54.6%)	901 (45.2%)	1588 (47.9%)	3248 (39.3%)	15604 (43.3%)	206781 (51.7%)
Divorced	38124 (12.9%)	4808 (16.1%)	1134 (16.8%)	666 (6.6%)	184 (9.2%)	482 (14.5%)	1256 (15.2%)	4148 (11.5%)	51751 (12.9%)
Widowed	35160 (11.9%)	3286 (11.0%)	691 (10.2%)	450 (4.5%)	138 (6.9%)	356 (10.7%)	643 (7.8%)	1840 (5.1%)	43454 (10.9%)
Separated	3915 (1.3%)	1394 (4.7%)	220 (3.3%)	127 (1.3%)	56 (2.8%)	92 (2.8%)	215 (2.6%)	1741 (4.8%)	7930 (2.0%)
Never married	42304 (14.4%)	9846 (32.9%)	1837 (27.2%)	3041 (30.2%)	590 (29.6%)	616 (18.6%)	2378 (28.8%)	9234 (25.6%)	71198 (17.8%)
A member of an unmarried couple	9579 (3.3%)	841 (2.8%)	356 (5.3%)	216 (2.1%)	114 (5.7%)	109 (3.3%)	469 (5.7%)	3231 (9.0%)	15169 (3.8%)
Missing	1671 (0.6%)	258 (0.9%)	78 (1.2%)	79 (0.8%)	11 (0.6%)	74 (2.2%)	58 (0.7%)	280 (0.8%)	3613 (0.9%)
Healthcare access									
Yes	276949 (94.0%)	26654 (89.0%)	6095 (90.2%)	9237 (91.6%)	1716 (86.1%)	2939 (88.6%)	7468 (90.3%)	26940 (74.7%)	365862 (91.5%)
No	17753 (6.0%)	3289 (11.0%)	665 (9.8%)	844 (8.4%)	278 (13.9%)	378 (11.4%)	799 (9.7%)	9138 (25.3%)	34034 (8.5%)

Racial bias assessment

**Figure 1:**
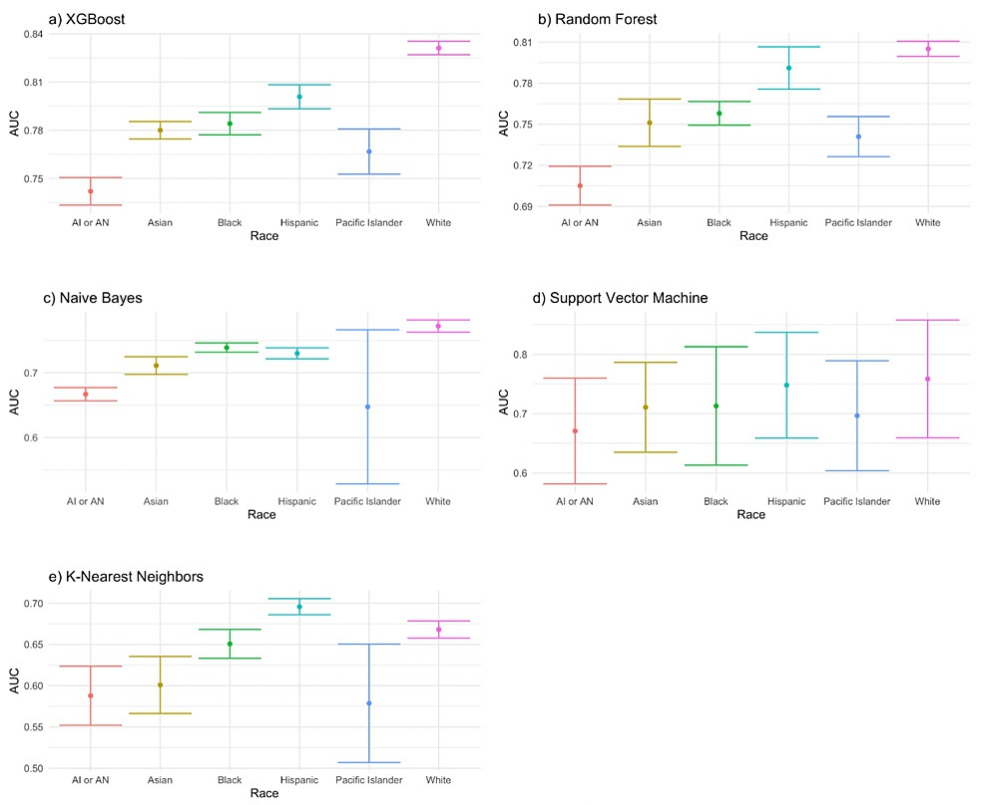
Race-specific performance for each machine learning algorithm Intervals represent 95% confidence intervals created from 20 iterations of bootstrapping the analysis AI or AN: American Indian or Alaskan Native

With only a few exceptions, we found that the performance for the White group was, in general, significantly higher than that of any other racial group across all ML algorithms. For the XGBoost algorithm, the most accurate ML algorithm in our analysis, the performance for the White group was statistically significantly higher than any other racial group. For the random forest algorithm, the next most accurate algorithm, the performance for Whites was significantly greater than all other groups except for the Hispanic group (although the point estimate for the White group was still greater than for the Hispanic group). Using the naive Bayes algorithm, the point estimate of the performance for the White group was higher than all other groups and this comparison was statistically significant for all groups except the Pacific Islander group. For SVM, the performance for the White group had the highest point estimate, although it was not statistically significant in terms of comparison with all the other groups. In KNN, the worst-performing algorithm in our analysis, the performance for the Hispanic group was significantly better than for the White group; however, the performance for the White group was still higher than for any other racial group - and the difference was significant when compared to every group except for the Black group. Figure 1 and Table 2 present the full results of our racial bias assessment for each ML algorithm.

Comparative performance of machine learning algorithms

XGBoost had the highest AUC of any ML algorithm for the prediction of healthcare access with race-specific average AUCs ranging from 0.74 to 0.83 (averaged across 20 iterations). The next highest in performance was the random forest algorithm, which had race-specific average AUCs of 0.71-0.81. The rest of the algorithms had lower performances with race-specific average AUC ranges of 0.65-0.77 (naive Bayes), 0.67-0.75 (SVM), and 0.58-0.70 (KNN). Table 2 shows the full list of race-specific performance metrics for each ML algorithm.

**Table 2:** Race-specific performance for each machine learning algorithm (mean AUC and 95% confidence intervals) Point estimate AUCs lower than that of the White group are bolded while those that are higher are italicized. Racial groups whose AUC is statistically significantly lower than the White group at an alpha of 0.05 are labeled with an asterisk (*). Confidence intervals are at the 95% level and were created from 20 iterations of bootstrapping the analysis

	XGBoost	Random forest	Naive Bayes	Support vector machine	K-nearest neighbors
American Indian or Alaskan Native	0.742 [0.733, 0.751]*	0.705 [0.691, 0.719]*	0.667 [0.675, 0.677]*	0.671 [0.582, 0.760]	0.588 [0.552, 0.624]*
Asian	0.780 [0.775, 0.785]*	0.751 [0.734, 0.768]*	0.711 [0.698, 0.725]*	0.711 [0.635, 0.786]	0.601 [0.566, 0.635]*
Black	0.784 [0.777, 0.791]*	0.758 [0.749, 0.767]*	0.739 [0.732, 0.746]*	0.713 [0.613, 0.813]	0.651 [0.633, 0.668]
Hispanic	0.801 [0.793, 0.808]*	0.791 [0.776, 0.807]	0.730 [0.721, 0.738]*	0.748 [0.659, 0.837]	0.696 [0.686, 0.705]*
Pacific Islander	0.767 [0.753, 0.781]*	0.741 [0.726, 0.756]*	0.647 [0.529, 0.766]	0.697 [0.604, 0.789]	0.579 [0.507, 0.650]*
White	0.831 [0.827, 0.835] (Ref)	0.805 [0.800, 0.811] (Ref)	0.772 [0.763, 0.781] (Ref)	0.759 [0.659, 0.858] (Ref)	0.668 [0.658, 0.678] (Ref)

## Discussion

In our study with an a priori-specified ML plan with minimal racial bias mitigation, we found overall higher model performance for the White group compared to all other racial groups across all five ML algorithms. Bootstrapping our analysis, we can visualize that this difference in performance between the White group and all other racial groups was, for most algorithms, statistically significant. Even using public data and traditional ML methods and packages in this project, our study illustrates the predictive perils of incorporating minimal racial bias mitigation, resulting in predictive disparities. While we did not directly study the underlying reason for this predictive discrepancy, the explanation is most likely multifactorial with the most dominant reason potentially being the fact that the majority of the training data consists of individuals from the White group. Perhaps a more representative training set would yield more equitable models. Other possible contributing factors include historical and measurement bias in the pre-processing phase stemming from historical racial inequities affecting health, healthcare access, and participation in research.

Secondarily, we found that XGBoost was the overall best prediction algorithm for our application with random forest following and the other algorithms following still. We a priori expected XGBoost to outperform the other models. XGBoost is a relatively newer, more powerful algorithm that has been widely successful and shown to outperform many other models in a variety of settings [21]. Next, the lack of significant predictive differences for SVM seems less to do with closer point estimates but rather wider variances of the models. The wider variances of these models suggest the tendency of SVM to produce more variability in its models; however, the underlying reason is not entirely clear. Perhaps, the size of the training data set is also a factor in performance variability; for the naive Bayes analysis, the Pacific Islander group was the smallest group in our data set and produced models with the largest variances compared to the other racial groups. Additionally, the one outlier in our results is the fact that the KNN algorithm predicted best for the Hispanic group (given that all the other algorithms predicted best for the White group). The reason for this is unclear and could reflect random chance or the nature of the KNN algorithm. The KNN algorithm works by classifying observations based on the status of those with best matching covariates (“neighbors”). There may be more homogeneity in the covariates of those in the Hispanic group without healthcare access; further, the Hispanic group also had the highest rates of our outcome - lack of healthcare access - compared to other racial groups (25.3%).

This study fits in with existing literature suggesting the prevalence of racial bias and predictive disparities in the performance of healthcare-related ML algorithms [11,12]. This is particularly concerning given recent literature showing that even when racial bias assessments are done, no or minimal resulting bias mitigation is performed [15].

The implications of this research are manifold. While ML in healthcare has seen a dramatic rise, guidelines and conversations regarding the assurance of equity of these models have lagged behind. Given the rise of ML and the importance of bias-resistance models across social lines, there needs to be more conversation, research, and guidelines surrounding methods for racial bias assessment and mitigation in models currently used and those in development.

Limitations

Several factors limited the predictive accuracy of the models created in this analysis. Significant levels of non-viable variables and missing data were both limitations for the models created in this analysis; however, we were able to limit this concern with our large sample size and use of multiple imputations. Another limitation was the relatively low prevalence of our outcome (lack of healthcare access), which can lead to models with increased specificity at the expense of sensitivity; however, using AUC as our performance metric affords a more comprehensive metric taking into account varying levels of sensitivity and specificity. Also, we could have used a wider range of tuning parameters for the ML models; however, we did try many different ML methods.

## Conclusions

Our study illustrates the racial bias that can result when creating ML models without proper bias mitigation. Healthcare-related ML models, both those currently being used and those in development, must incorporate robust racial bias assessment and mitigation methods. Only through crafting fair models can ML, a powerful tool, be a powerful force for promoting equitable healthcare for all.
